# Some Like It Rock ‘N’ Cold: Speleomycology of Ravništarka Cave (Serbia)

**DOI:** 10.3390/jof11100706

**Published:** 2025-09-29

**Authors:** Miloš Stupar, Željko Savković, Marija Pećić, Dragana Jerinkić, Olga Jakovljević, Slađana Popović

**Affiliations:** Faculty of Biology, University of Belgrade, Studentski Trg 16, 11000 Belgrade, Serbia; zsavkovic@bio.bg.ac.rs (Ž.S.); marija.pecic@bio.bg.ac.rs (M.P.); d.predojevic@bio.bg.ac.rs (D.J.); olga.jakovljevic@bio.bg.ac.rs (O.J.); or spopovic.bio@gmail.com (S.P.)

**Keywords:** cave, fungi, psychrophiles, speleobiology

## Abstract

Caves and other subterranean ecosystems are characterized by stable, low temperatures, high humidity, and limited nutrient input, creating unique environments for extremophilic microorganisms. Among them, fungi play key roles in organic matter degradation, mineral interactions, and biogeochemical cycling, yet the diversity and adaptations of cold-adapted fungi in cave habitats remain insufficiently explored. This study investigated psychrophilic and psychrotolerant fungi inhabiting the stone surfaces of Ravništarka Cave in Eastern Serbia. Biofilm samples were collected from nine sites and analyzed using culture-based isolation on both nutrient-rich and diluted media, followed by incubation at 10 °C, 25 °C, and 37 °C. Fungal identification combined morphological characteristics with molecular analyses of the ITS region and *BenA* gene, while ecological roles were assigned using FUNGuild. A total of 41 fungal species were documented, spanning Ascomycota (53.1%), Basidiomycota (43.7%), and Mortierellomycota (3.1%) phyla. The genus *Penicillium* exhibited the greatest species richness, with 14 taxa documented, including *P. chrysogenum*, *P. glandicola*, and *P. solitum*, all previously associated with cold or oligotrophic environments. The psychrotolerant species *Mortierella alpina* was the only representative of Mortierellomycota. Ecological guild assignment revealed fungi functioning at different trophic levels, highlighting their multifunctional ecological roles in extreme subterranean habitats.

## 1. Introduction

Ecosystems like caves or human-made underground facilities maintain stable and low temperature conditions and exhibit low nutrient availability with limited energy input throughout the year [1], and as such, facilitate the growth of extremophilic microorganisms, including oligotrophs and psychrophiles or psychrotolerant species (psychrotrophs) [2]. In spite of conditions different from epigean environments, caves are becoming “hotspots for microbial diversity” due to the presence of both described and yet-to-be-discovered species, among which fungi are among the most dominant groups of microorganisms in the caves [3,4]. Speleomycology is an emerging scientific field that examines the diversity, ecology, and function of cave-dwelling fungi [5]. Fungi contribute significantly to biogeochemical processes within cave environments, particularly in the degradation of organic material and interactions with mineral surfaces. In subterranean systems, stone surfaces offer a widespread but challenging habitat for microbial colonization due to limited nutrients and extreme physical and chemical conditions. Yet, lithobiontic fungi have been detected on a range of cave substrata. Furthermore, subaerial biofilms (SAB), which frequently grow on cave walls and ceilings, are composed of fungi, among other microbial components. In addition to precipitation and the development of corrosion residues, the metabolic activity of SAB-forming fungi may cause weathering of stone substrata and unique colorations on speleothems [6]. In fact, the biological breakdown of rocks, which includes physical particle separation and the release of organic acids and secondary metabolites, is started by the development of fungi on mineral surfaces [7,8]. Because of this, fungi can be categorized as microorganisms that have a major impact on biogeochemical processes, which undoubtedly contribute to the soil’s enrichment and diversity [9,10]. Despite their ecological importance, the diversity and adaptation strategies of cold-adapted fungi in subterranean habitats remain understudied [4].

Psychrophilic and psychrotolerant fungi have evolved adaptations that allow them to survive and remain metabolically active at low temperatures. These adaptations enable them to colonize cold habitats, including permafrost, alpine soils, and cave systems [11]. Psychrophilic fungi are specialized to grow at low temperatures—at or near 0 °C [12]—and are well-adapted to permanently cold environments such as glaciers, Arctic and Antarctic soils, and deep caves [4,13,14]. More precisely, maximum growth for psychrophiles is achieved by optimal temperatures lower than 20 °C [15]. On the other hand, psychrotolerant fungi are not cold specialists, and they have optimal growth between ~20 and 30 °C; however, they can survive and remain active in cold environments such as caves, alpine soils, or food storage [16]. According to Russel [17,18], “true” or “obligate” psychrophiles can be categorized as stenothermal, while psychrotolerants can be categorized as eurythermal. This definition accounts for the fact that microorganisms that are cold-adapted and have a wide range of growth temperatures are far more prevalent in cold climates, most likely due to their ability to withstand a wide range of temperatures. Even though the ideas of eurythermal and stenothermal psychrophiles appear to be more succinct, traditional ideas are still extensively used [19].

Despite increasing interest in subterranean fungi, few studies have specifically addressed the diversity of cold-adapted fungi inhabiting stone surfaces in cave ecosystems [11,20]. Understanding these communities is crucial for elucidating microbial adaptation and ecological functioning in extreme environments. Therefore, this study aimed to isolate and characterize psychrophilic and psychrotolerant fungi colonizing stone substrata in Ravništarka Cave in Eastern Serbia, using culturing techniques combined with morphological and molecular identification. The findings contribute to our understanding of fungal diversity and adaptation in cold subterranean habitats.

## 2. Materials and Methods

### 2.1. Sampling Location

The village of Ravnište, close to Kučevo, Serbia, is home to the Ravništarka Cave, formerly known as the “Sava’s Cave” (44°24′30″ N, 21°37′04″ E). In 1894, renowned Serbian geologist Jovan Cvijić entered the accessible entry section of Ravništarka Cave (60–80 m). But nearly a century later, in 1980, two local boys made the final discovery of the cave after passing through the cave channels entirely. Speleologists then visited the cave and followed the children’s journey. The elevation of the cave entrance is 406.6 m. The length of the tourist path through the cave is 550 m, while the main channel is 501.5 m long. The overall length of all cave channels is above 600 m. The Ponorac stream, which starts 2 km upstream in the much smaller Bisa Cave, flows through the active river cave of Ravništarka. The cave’s main channel, which resembles a vast tunnel, is one of its most notable characteristics. It is elaborately decorated with a variety of cave structures on the walls and ceilings, including Glavonje, Lepa Ravništarka, and Šarac Kraljevića Marka. Sava’s Canal, Youth Canal, Swan Lake, The White Castle, Leopard Canal, The Black Castle (a hall), Source Channel, Little White Castle, and Dušan’s galleries are the portions that make up the cave. Every area of the cave is distinct, one-of-a-kind, and full of formations that are valuable to view and preserve. In 2007, Ravništarka Cave was fully prepared for tourism after being designated a Protected Natural Resource, or Natural Monument (https://www.tokucevo.org/pecina-ravnistarka/, accessed on 10 June 2025).

### 2.2. Ecological Parameters Measurement and Organic Matter Analysis

Cave microclimatic parameters, the temperature (T (°C) and relative humidity (RH (%)) in the vicinity of sampling points, were measured with the Temperature Humidity Meter (Extech, Nashua, NH, USA). Five measurements of each microclimatic parameter were made at each site, and then the mean values were determined. In addition, the biofilm was sampled using circular metal molds spanning a predetermined area in order to conduct organic matter analysis [21].

### 2.3. Sampling of Rock Surface Associated Mycobiota

**Figure 1:**
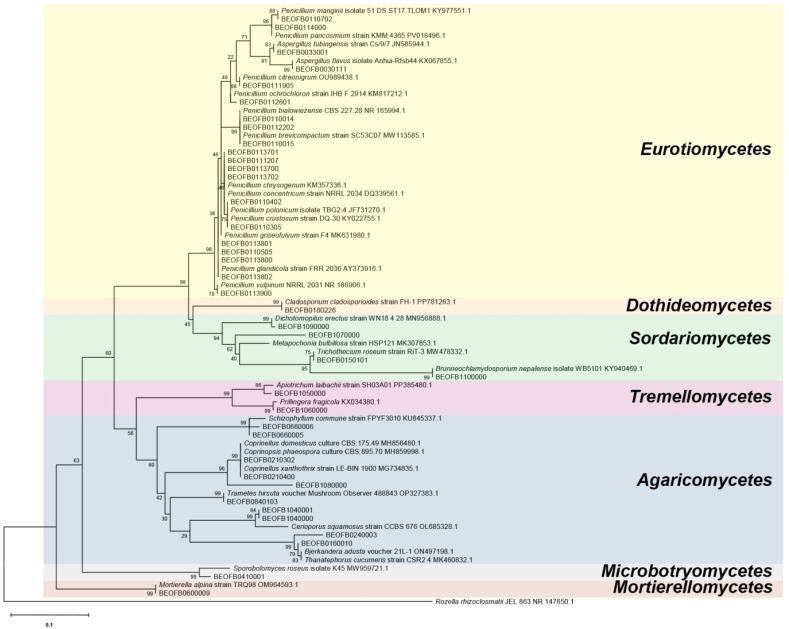
ITS region maximum likelihood cladogram of the fungal isolates from Ravništarka Cave.

In order to isolate fungi from stone cave substrata, five samples were collected with sterile cotton swabs that were brushed over the biofilm surface (~10 cm^2^) from each of the nine sampling points (R1: Sava’s Channel, R2: Youth Channel—Rudonja, R3: Youth Channel—between the Tufa terrace and the Curtain, R4: Swan Lake—between the Swan and the Lake Fairies, R5: Swan Lake—Beautiful Ravništarka, R6: The White Castle—Snow White’s Balcony, R8: Black Castle—near Ravništarci, and R9: Little White Castle—above the stream; the exact position of the sampling sites can be seen in the Figure 1 of the Popović et al. [21]. Afterwards, swabs were placed in sterile plastic bags upon sampling prior to laboratory processing. For the purpose of this study, sampling was conducted once, in May 2023, in contrast to the work performed in Popović et al. [21], when the sampling was conducted twice.

### 2.4. Fungal Isolation

Swabs were inoculated on solidified nutrient media in a zigzag pattern by applying the streak plate method. Potato Dextrose Agar (PDA, BIOLIFE) medium was used as a non-selective isolation medium. In order to isolate fungi adapted to low nutrient conditions, swabs were inoculated on Diluted Malt Extract Agar (DMEA, 1:20, NEOGEN culture media). Inoculation on both media used has been done in triplicate. Following inoculation, PDA and DMEA plates were incubated at three distinct temperatures: 12 ± 2 °C (which is indicative of the cave conditions—“cave temperature”) to isolate psychrotholerant and/or psychrophilic fungi; 25 ± 2 °C (“room temperature”) to isolate mesophilic fungi; and 37 ± 2 °C (“human body temperature”) to isolate thermophiles and human opportunistic pathogens.

### 2.5. Fungal Identification

The presence of particular micromorphological structures, such as conidia, conidiophores, and spore-bearing bodies, as well as the color and growth pattern of the colonies, was considered for preliminary phenotypic identification of cave isolates. For microscopy inspection of obtained isolates, a stereomicroscope (Stemi DV4, Carl Zeiss, Oberkochen, Germany) and a light microscope (Zeiss Axio Imager M.1) were applied. In that sense, the identification keys of Watanabe [22], Samson et al. [23], Visagie et al. [24], Varga et al. [25], Bensch et al. [26], Shao et al. [27], and Frisvad et al. [28] were used.

Molecular techniques are employed to identify non-sporulating isolates and to validate initial phenotypic identification. Thus, the PDA and DMEA media were reinoculated with primary fungal isolates, and they were cultured for a week at the original isolation temperature (“cave, room or human body”). Dry peripheral mycelia of reisolated cultures (about 40 mg) were harvested for DNA extraction in accordance with the directions provided by the Quick-DNA Fungal/Bacterial Miniprep Kit (ZYMO RESEARCH USA, Irvine, CA, USA). As previously described by Savković et al. [29], PCR amplification of the ITS region and *BenA* gene was performed using specific ITS1/ITS4 [30] and Bt2a/Bt2b primers [31], respectively. In a 0.5× TBE buffer, the amplified DNA fragments were separated in 1% agarose gels. DNA presence has been confirmed by using Midori Green stain under UV light [29]. Following that, the PCR products were sent to Macrogene (Amsterdam, The Netherlands) for purification and sequencing. The BLAST tool (BLAST+ 2.7.1 of the NCBI) was then used to compare the obtained sequences with additional comparable sequences that had been deposited to the National Center for Biotechnology Information (NCBI). Ultimately, the acquired fungal DNA sequences were deposited in the NCBI GenBank database.

### 2.6. Phylogenetic Analysis

Sequence alignment was carried out with the MEGA11 software’s CLUSTALW alignment [32]. The phylogenetic tree was constructed using maximum likelihood phylogeny (1000 bootstrap repetitions) based on the alignment and comparison of DNA sequences. Kimura’s 2-parameter model was determined as the best for estimating genetic distances between tested sequences, measured in terms of nucleotide substitutions per site. *Rozella rhizoclosmatii* strain JEL 863 was used as the outgroup.

### 2.7. Ecological Function Assessment of Fungal Isolates

The FUNGuild v1.0 program (Guilds_ v1.1.py script, database: http://www.stbates.org/funguild_db.php (accessed on 1 May 2025)) [33], along with additional literature data [34,35,36,37,38,39,40,41,42], was used to sort all fungal isolates according to their ecological function. Species were categorized according to their trophic mode into the following ecological groups: pathotroph (P), saprotroph (S), and symbiotroph (Sy). Furthermore, according to their trophic mode, species were sorted into the following guilds: animal pathogen (ap), endophyte (en), epiphyte (ep), fungal parasite (fp), lichen parasite (lp), litter saprotroph (ls), soil saprotroph (ss), dung saprotroph (ds), plant pathogen (pp), undefined saprotroph (us), and wood saprotroph (ws).

To check the geographical distribution and substrate preference of the obtained fungal isolates with other records of cave mycobiota worldwide, a list acquired through a comprehensive NCBI database search and presented in a review by Savković et al. [4] was used as a comparison checklist.

### 2.8. Multivariate Analyses

The fungal isolates documented in Ravništarka Cave were grouped into genera using the “train averages” option in the Canoco software 5.0 [43] and related separately to the following factors: ecological parameters measured in the cave (T and RH), cultivation temperatures, guilds, trophic level, and substrates (for the last three, data from the literature were used). Principal component analysis (PCA) and redundancy analysis (RDA) were used for these purposes.

## 3. Results

Speleomycological analyses, along with measurements of ecological parameters and organic matter, were conducted at nine sampling sites within the Ravništarka cave. Temperature varied among the sampling sites and ranged from 10.8 to 13.1 °C. Relative humidity rose from site R1 to site R9 as the tunnel cave’s depth climbed, and a great value was recorded for R9 (92%). Each sampling site had a different amount of organic materials (0.17 mg cm^−2^ in R5, 3.37 mg cm^−2^ in R9). The results of ecological parameters (T, RH) and organic matter content were presented in the publication of Popović et al. [21].

Classical mycological analysis, along with molecular techniques employed on fungal representatives, led to the isolation of 49 cave fungal dwellers and to the identification of 41 different species. Isolated fungi belonged to three phyla. The majority of identified taxa were members of Ascomycota (53.13%) and Basidiomycota (43.75%), while *Mortierella alpina* was the only representative identified from the phylum Mortierellomycota (3.13%). All the species obtained in the study were filamentous fungi, except *Prillingera fragicola* and *Sporobolomyces roseus*, which are representatives of basidiomyceteous yeasts. The highest species richness was documented for the genus *Penicillium*, with 13 species identified (*P. bialowiezense*, *P. brevicompactum*, *P. chrysogenum*, *P. citreonigrum*, *P. concentricum*, *P. dierckxii*, *P. expansum*, *P. glandicola*, *P. griseofulvum*, *P. manginii*, *P.ochrochloron*, *P. solitum*, and *P. vulpinum*). The fungal ITS rDNA nucleotide sequences obtained in the study were submitted to GenBank under accession numbers from PV871528 to PV871567. Furthermore, *BenA* sequences used for the identification of *Aspergillus* and *Penicillium* species with higher taxonomic resolution were submitted under accession numbers PV893141, PV893142, PV936447–PV936452, PX061017, PX060351, and PX099057–PX099060 (Table 1).

A phylogenetic tree of the obtained isolates based on the neighbor-joining phylogeny of the ITS region is presented in Figure 1. *Aspergilli* and *Penicillia* formed a well-supported Eurotiales clade (bootstrap value, bp = 98). Within the phylum Ascomycota, two more clades were present: Dothideomycetes with members of the genus *Cladosporium* (bp = 99) and Sordariomycetes with members of the genera *Metapochonia*, *Brunneochlamydosporium*, and *Trichothecium* (bp = 94). Likewise, three clades formed within the Basidiomycotaphylum: Tremellomycetes with members of the genera *Apiotrichum* and *Prillingera* (bp = 99), Agaricomycetes with members of *Coprinellus*, *Schizophyllum*, *Cerioporus*, *Thanatephorus*, *Bjerkandera*, and *Trametes* genera (bp = 60), and Microbotryomycetes with *Sporobolomyces roseus* (bp = 99), *Mortierella alpina* clustered within the Mortierellomycetes clade (bp = 99).

**Figure 2:**
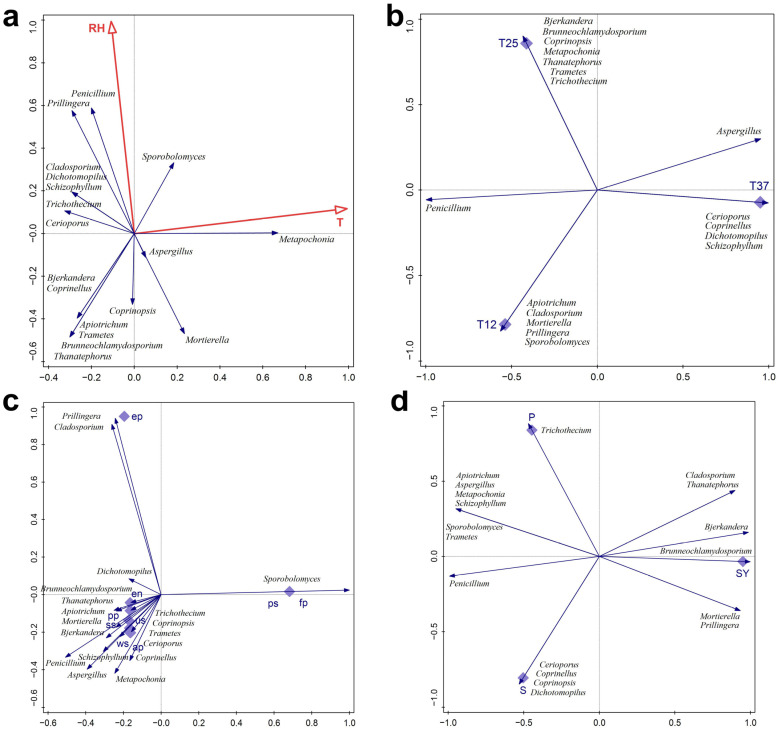
(**a**) RDA of fungi isolated from Ravništarka Cave in relation to ecological parameters measured in cave—T and RH, (**b**) PCA of fungi in relation to cultivation temperature, (**c**) PCA of fungi in relation to guilds (ap—animal pathogen; en—endophyte; ep—epiphyte; fp—fungal parasite; pp—plant pathogen; ps—plant saprotroph; ss—soil saprotroph; us—undefined saprotroph; ws—wood saprotroph), (**d**) PCA of fungi in relation to trophic level (P—pathotroph; S—saprotroph; Sy—symbiotroph).

Fungi were also related to T and RH measured in caves (RDA, Figure 2a). Several genera were positively correlated with RH (all genera in the upper left part of the ordination diagram), with *Penicillium*, *Prillingera*, and *Sporobolomyces* being the most pronounced. With respect to T, a positive correlation with this factor was observed for *Metapochonia* and *Sporobolomyces* (Figure 2a).

When observing the temperature at which fungi were cultivated in relation to fungi that are recorded in this study, three groups are clearly separated on the PCA ordination diagram (Figure 2b). *Apiotrichum*, *Cladosporium*, *Mortierella*, *Prillingera*, and *Sporobolomyces* are related to cultures grown at cave temperature; *Bjerkandera*, *Brunneochlamydosporium*, *Coprinopsis*, *Metapochonia*, *Thanatheporus*, *Trametes*, and *Trichothecium* were isolated at room temperature, while *Cerioporus*, *Coprinellus*, *Dichotomophilus*, and *Schyzophylum* were cultured exclusively at human body temperature (Figure 2b). Fungi of the genus *Penicillium* were obtained from cultures grown at both 12 °C and 25 °C, while *Aspergillus* species were isolated at 25 °C and 37 °C. The majority of cave-dwelling fungi were isolated on “room temperature” (46.94%), followed by “cave temperature” (30.61%). Furthermore, only 12.24% of fungal species were isolated using DMEA.

The PCA ordination diagram shows that many fungal representatives and many guilds are grouped in the lower left part of the ordination diagram. Notable are *Sporobolomyces*, the only genus related to fungal parasites and plant saprotrophes, as well as *Cladosporium* and *Prillingera*, which are the only ones related to the epiphyte guild (Figure 2c).

The documented fungi were at different trophic levels (Figure 2d). *Cerioporus*, *Coprinellus*, *Coprinopsis*, and *Dichotomophilus* are saprothrops; *Trichothecium* is pathothroph, while the representative *Brunneochlamydosporium* is symbyotroph. The rest of the fungal genera recorded had two trophic levels, with the exception of *Bjerkandera* and *Penicillium*, which had three (S, P, and Sy).

**Figure 3:**
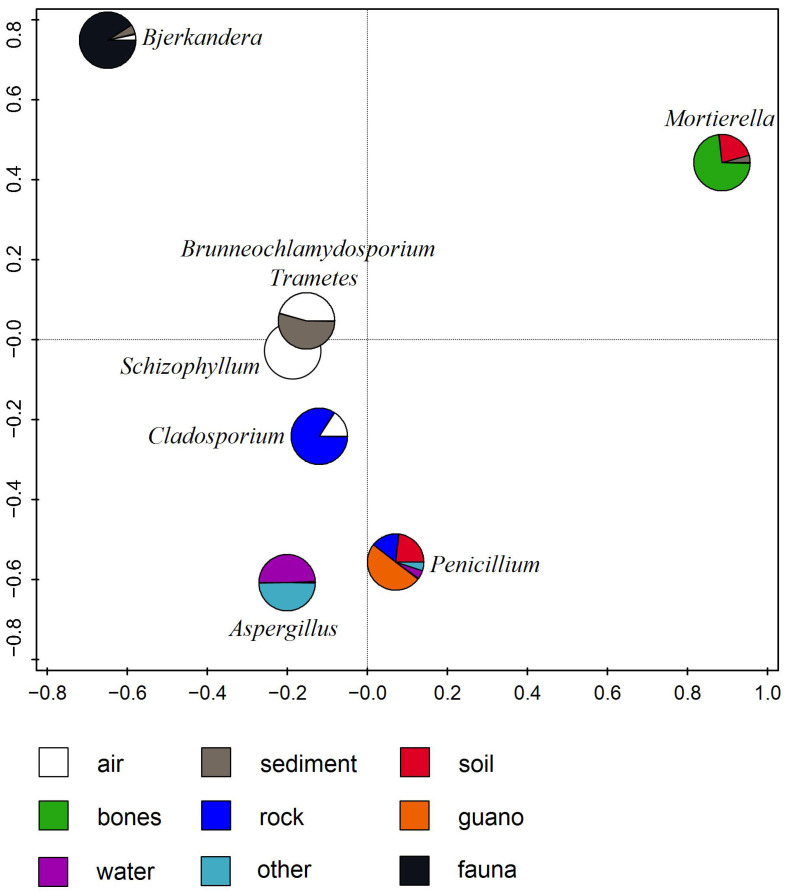
RDA of fungi isolated from Ravništarka Cave in relation to substrate (based on Savković et al. [4]).

Although the fungi in this study were isolated from SAB developed on stone substrate, the representatives were also associated with the different substrates on which they occur according to the literature [4] (for which we did not have data on all genera). According to Figure 3, *Bjerkandera* is a genus mainly found on fauna, *Mortierella* on bones of various animals, and *Brunneochlamydosporium* and *Trametes* in sediment and air, while *Schyzophilum* was only detected in air. *Cladosporium* was mainly found on rocks, and *Aspergillus* in water, while representatives of *Penicillium* were found on many different substrates, but mainly on guano.

## 4. Discussion

The speleomycological studies remain an area in which many aspects of cave-dwelling fungal communities are not yet fully understood, particularly with regard to the diversity of rock-inhabiting and psychrophilic fungi. Due to their oligotrophic nature and constant low temperature, caves are recognized as a habitat for a variety of microorganisms, including fungi that have evolved to harsh environments. In the research presented here, the highest species richness was documented for the members of the phylum Ascomycota, which is in accordance with several speleomycological reviews [3,4,44]. In fact, Ascomycota members always have a higher documented diversity than other fungal phyla (Basidiomycota, Mucoromycota, and Mortierellomycota) when fungi are isolated from a variety of substrates, particularly in harsh environmental settings like caves [4], deserts [45], deep-sea habitats [46], and even Antarctic soils [47]. Numerous ascomycetes can also exist as endolithic or epilithic fungi [48] and form SABs on different stone substrates in the caves, including stalactites, stalagmites, and cave walls [49]. The oligotrophic conditions found in caves, in particular, encourage the synthesis of unique antimicrobial compounds, which are crucial for finding novel treatments in microbial niches that are competitive [8]. Similarly, fungal species adapted to cold environments are the most successful eukaryotic extremophiles, and as such, they are potential producers of cold-active enzymes, metabolites with various biological activities, and exopolysaccharides [11]. Hence, speleomycological studies dealing with the diversity of rock-dwelling cave fungi, oligotrophs, psychrophils, and psychrotolerants are not only of ecological significance but are also important as a source for novel biotechnological and pharmaceutical compounds.

The highest species richness was documented for the members of the genus *Penicillium* (14 identified species), which is also in accordance with the literature data [4]. Low temperatures, along with nutrient-poor conditions, are limiting environmental factors present in the caves that many *Penicillium* species can withstand [50]. Additionally, some *Penicillium* species are xerotolerant and show resilience to desiccation [51]. Furthermore, some species of this genus can degrade limestone substrata, including cave walls; hence, their presence on rock substrates cannot be disregarded. According to Sternflinger [52] and Burford et al. [53] the *Penicillium* species can affect rocks through biochemical degradation and the secretion of organic acids, oxidation of Fe(II) and Mn(II), adsorption of Al, Zn, Cd, U, Th, Pb, and Sn, solubilization of rock phosphate and coal, reduction of Fe(III), and mineralization of materials like halloysite, montmorillonite, or todorokite. *P. chrysogenum* isolated from sampling site R3 at “cave temperature” in this research is, according to the NCBI database search checklist published by Savković et al. [4], among the most frequently encountered fungal species within the cave environment and is isolated so far from various subterranean substrata (i.e., rock, sediments, guano, water, air, soil, sludge…). Ogórek et al. [7] reported *P. chrysogenum* as a fungus that most frequently occurred on the rock surface in Driny Cave in Slovakia, which was isolated in laboratory conditions at 15 °C. Furthermore, Pusz et al. [54] reported the presence of *P. chrysogenum* on rock surfaces in Jarkowicka Cave (Poland). Other studies also confirm the psychrotrophic nature of *P. chrysogenum*. Chen et al. [55] isolated *P. chrysogenum* strain A096 from an Arctic sediment sample. These authors also purified and characterized an antifungal protein (“Pc-Arctin”) obtained from this cold-environment strain. Arctic-derived *P. chrysogenum* is a producer of dimeric tetrahydroxanthones, compounds with antimicrobial activity [56]. Glodowsky et al. [57] reported Antarctic strains of *P. chrysogenum* as the new source for cold-active transglutaminase. Kozlovskii et al. [58] isolated strains from *Penicillium* section *Chrysogena* (which includes *P. chrysogenum*/*P. rubens*) from permafrost deposits, frozen volcanic ash, and Antarctic lake water and reported the presence of exometabolites belonging to penicillins, chrysogines, roquefortines, xanthocillins, and simple tryptophan derivatives.

In this study, the most frequently encountered *Penicillium* species was *P. glandicola*. This species was documented on three sampling sites (R4, R8, and R9) and was isolated at both cave and room temperatures. Ogórek et al. [59] reported *P. glandicola* as the main “culprit” responsible for dark stains on rock surfaces in Driny Cave (Slovakia). *P. glandicola* is a psychrotrophic fungus, able to form viable mycelium after storage at −72 °C, to show active growth at temperatures from 5 to 20 °C but without conidia germination, and without active growth at “human body temperature” [59]. One distinctive morphological feature of *P. glandicola* is the formation of coremia, the research of which is presented here, documented using PDA medium. *P. solitum* is a species well known for causing blue mold on apples and other stored fruits under refrigeration, which is why it is frequently isolated from cold storage environments; however, due to the optimum growth range in the mesophilic zone (~20–25 °C), it is considered psychrotolerant [23]. Mitova et al. [60] reported *P. solitum* on the rock substratum of Palaeolithic paintings of Magura Cave in Bulgaria. Furthermore, Vanderwolf et al. [61] reported *P. solitum* on the fur and skin of healthy, hibernating bats, *Myotis lucifugus* and *M. septentrionalis* in New Brunswick caves (Canada). The sole *Penicillium* species in this research isolated on DMEA was *P. concentricum*. This fungus can grow on substrates with low nutrient availability, such as stored grain surfaces, wooden packing materials, and even nutrient-poor agar media, which makes it oligotrophic. Poli et al. [62] reported this species on sediments in the Costacalda cave (Maritime Alps) in Italy.

*Mortierella alpina* is the only member of the phylum Mortierellomycota documented in this research. This species is isolated at cave temperature from sampling site R2. *M. alpina* is regarded as a psychrotrophic species that grows well at low temperatures (around 5–10 °C) and hence can be frequently isolated from soils in temperate and cold environments. For some strains of *M. alpina* isolated from the Antarctic region, the production of arachidonic and linoleic acid, along with the change of fatty acid composition in the cell membrane, has been proven as the mechanism of adaptation to low temperatures [63]. The members of the genus *Mortierella*, including *M. alpina*, are frequently isolated from the caves worldwide [4]. To name a few reports, *M. alpina* has been isolated from sediments in Heshang Cave in China [64], bones of Paleolithic bear *Ursus spelaeus* in Niedźwiedzia Cave (Kletno) in Poland [65], and sediments from the show cave Castanar Ibor in Spain [66].

The majority of fungal species isolated at “human body temperature” in this research were basidiomycetes. A subset of basidiomycetes can grow or survive at temperatures close to mammalian body temperature; some of them inhabit warm microhabitats such as compost piles, decaying organic matter in sunny environments, or deep soil layers where microbial metabolism raises the temperature above the ambient [67]. Furthermore, some basidiomycetes are pathogenic or opportunistic species adapted to mammalian body temperature. For example, *Schizophyllum commune*, an opportunistic human and animal pathogen linked with several infections mostly affecting the respiratory system, can grow at higher temperatures. To name a few case studies, Oliveira et al. [68] reported rapid mycelial expansion in a thermotolerance test at 37 °C of *S. commune* bloodstream isolates from patients with fungemia in Brazil. Furthermore, Won et al. [69] successfully cultivated at human body temperature an *S. commune* isolate from a patient suffering from allergic fungal sinusitis. The reports of *S. commune* in caves are scarce; however, air sampling found *S. commune* to be among the most abundant airborne fungi in several chambers of the cave “Cueva del Tesoro” in Spain [70]. Culture-based surveys also confirmed the presence of *S. commune* in non-touristic halls of Le Stegodon Cave of Satun UNESCO Global Geopark in Thailand [71]. *S. commune* infections are very rare, and the presence of this fungus in the SAB developed on a cave stone represents a low risk to human visitors. Nevertheless, the presence of potential pathogens in show caves must not be neglected [72]. Both *Coprinellus* species in this research (*C. xanthothrix* and *C. domesticus*) are isolated at human body temperature. It should be noted that most coprinoid species have optimal mycelial growth around 20–30 °C and only a subset are thermotolerant enough to grow (sometimes poorly) at 37 °C. For both *Coprinellus* species, tolerance to the high temperatures has been proved in vitro, i.e., irregular growth at 40 °C was reported for *C. xanthothrix* and *C. domesticus* with a low growth rate [73]. Apart from the mentioned, there are no previous reports regarding the presence of these species on various substrata within the caves. However, *C. xanthothrix* has been reported as a rock-dwelling fungus isolated from the limestone of the old cathedral of Coimbra in Portugal [74].

A total of 10 species documented on rock substrate in the Ravništarka cave were not previously reported in caves worldwide. Initial reports for cave environments include *Aspergillus tubingiensis*, *Cerioporus squamosus*, *Coprinellus domesticus*, *C. xanthothrix*, *Coprinopsis phaeospora*, *Metapochonia bulbillosa*, *Penicillium manginii*, *Prillingera fragicola*, *Thanatephorus cucumeris*, and *Trichothecium roseum*. Despite the fact that the number of speleomycological investigations is continuously rising, caves are still regarded as ecologically poorly investigated environments. In that sense, studying the diversity of cave mycobiota can lead not just to first reports of fungal species previously not documented in caves, but also to the description of novel fungal taxa and to the detection of species with unique biochemical properties and potential biochemical applications [4].

*Dichotomopilus erectus* and *Aspergillus tubingiensis* were the only ascomycetes isolated at human body temperature in this study. Recent taxonomic and phylogenetic analyses reclassify *Chaetomium erectum* into *D. erectus* among species belonging to the Chaetomiaceae family that includes thermotolerant/thermophilic species [75]. *A. tubingiensis* is a member of the section *Nigri*, whose many strains can grow at 37 °C, and some tolerate up to ~40 °C, and hence should be regarded as a thermotolerant mesophile [25,76]. *A. tubingensis* is regularly reported from caves (air, sediment/soil, guano, and other cave substrates) in multiple culture-based surveys worldwide. For example, this species was documented from sediments in the Bossea cave (Maritime Alps) in Italy [62]. Additionally, air-and-sediment surveys in the Nerja Cave (Spain) and other karst caves report this species among the species recovered from cave air [77]. Furthermore, *A. tubingensis* in this study is recovered on DMEA, suggesting its oligotolerance. Apart from that isolate, other fungi recovered from this medium in this study were *Cerioporus squamosus*, *Metapochonia bulbillosa*, *Penicillium concentricum*, *Trichothecium roseum*, and *Coprinopsis phaeospora*. Due to the absence of available nutrients, oligotrophic fungi are frequently present in cave environments [78].

## 5. Conclusions

This study highlights the diversity of psychrophilic and psychrotolerant fungi colonizing stone surfaces in Ravništarka Cave, Serbia. A total of 41 species were identified, with *Penicillium* showing the greatest richness, confirming the adaptability of this genus to cold and oligotrophic environments. Our findings confirm that cave rock surfaces, despite nutrient limitations and harsh physicochemical conditions, sustain diverse fungal communities with important ecological functions in organic matter turnover and mineral interactions. Furthermore, these fungi represent a valuable reservoir of bioactive metabolites and cold-active enzymes with potential biotechnological applications. This work expands knowledge of cave-dwelling fungi and emphasizes the importance of further integrative studies to uncover their ecological roles and applied potential.

## Figures and Tables

**Table 1:** Mycobiota of stone surfaces in Ravništarka cave, with associated ecological guild and trophic mode. An asterisk indicates a first report in cave environments.

Species	Isolate Number	ITS Gene Bank Acc. No. (Query, E Value, Homology)	*BenA* Gene Bank Acc. No. (Query, E Value, Homology)	Guild	Trophic Mode
*Apiotrichum laibachii*	BEOFB1050000	PV871536 (100%, 0.0, 100.00%)	-	ap, us	P, S
*Aspergillus flavus*	BEOFB0030111	PV871564 (100%, 0.0, 99.82%)	-	ap, ws, pp	P, S
*Aspergillus tubingiensis* *	BEOFB0033001	PV871567 (97%, 0.0, 99.47%)	PV893141 (100%, 0.0, 100.00%)	pp, ss	P, S
*Bjerkandera adusta*	BEOFB0160010	PV871552 (100%, 0.0, 99.83%)	-	pp, en, ws	P, S, Sy
*Brunneochlamydosporium nepalense*	BEOFB1100000	PV871549 (100%, 0.0, 99.80%)	-	en	Sy
*Cerioporus squamosus* *	BEOFB1040000	PV871532 (100%, 0.0, 100.00%)	-	ws	S
	BEOFB1040001	PV871545 (100%, 0.0, 100.00%)	-	ws	S
*Cladosporium cladosporioides*	BEOFB0180226	PV871537 (100%, 0.0, 100.00%)	-	ep, en, pp	P, Sy
*Coprinellus domesticus* *	BEOFB0210302	PV871529 (100%, 0.0, 100.00%)	-	us	S
*Coprinellus xanthothrix* *	BEOFB0210400	PV871528 (100%, 0.0, 100.00%)	-	us	S
*Coprinopsis phaeospora* *	BEOFB1080000	PV871558 (100%, 0.0, 98.91%)	-	us	S
	BEOFB1080001	PV871559 (100%, 0.0, 98.91%)	-	us	S
*Dichotomopilus erectus*	BEOFB1090000	PV871546 (100%, 0.0, 99.02%)	-	us	S
Metapochonia bulbillosa *	BEOFB1070000	PV871550 (100%, 7 × 10^−128^ 97.13%)	-	ap, us	P, S
*Mortierella alpina*	BEOFB0600009	PV871534 (100%, 0.0, 99.84%)	-	ss, en	S, Sy
*Penicillium bialowiezense*	BEOFB0112202	PV871538 (100%, 0.0, 99.81%)	-	pp, ss	P, S
*Penicillium brevicompactum*	BEOFB0110014	PV871544 (100%, 0.0, 100.00%)	-	en	Sy
	BEOFB0110015	PV871547 (100%, 0.0, 99.01%)	-	en	Sy
*Penicillium chrysogenum*	BEOFB0111207	PV871535 (100%, 0.0, 100.00%)	-	pp	P, S
*Penicillium citreonigrum*	BEOFB0111905	PV871557 (93%, 0.0, 99.81%)	PX099057 (100%, 0.0, 100.00%)	ap	P
*Penicillium concentricum*	BEOFB0113700	PV871539 (na)	PV893142 (99%, 0.0, 100.00%)	us	S
	BEOFB0113701	PV871543 (na)	PV936450 (100%, 0.0, 100.00%)	us	S
	BEOFB0113702	PV871551 (na)	PV936451 (100%, 0.0, 100.00%)	us	S
*Penicillium dierckxii*	BEOFB0114000	PV871562 (na)	PX099060 (90%, 0.0, 100.00%)	ss	S
*Penicillium expansum*	BEOFB0113303	-	PX061017 (97%, 0.0, 100.00%)	pp	P
*Penicillium glandicola*	BEOFB0113800	PV871540 (100%, 0.0, 99.63%)	PV936448 (100%, 0.0, 99.76%)	ss	S
	BEOFB0113801	PV871542 (94%, 0.0, 99.81%)	PV936449 (100%, 0.0, 99.76%)	ss	S
	BEOFB0113802	PV871553 (100%, 0.0, 99.82%)	-	ss	S
*Penicillium griseofulvum*	BEOFB0110505	PV871563 (100%, 0.0, 99.63%)	-	ap	P
*Penicillium manginii* *	BEOFB0110702	PV871560 (100%, 0.0, 99.52%)	PX099058 (100%, 0.0, 100.00%)	ss	S
*Penicillium ochrochloron*	BEOFB0112601	PV871554 (100%, 0.0, 99.11%)	PX060351 (100%, 0.0, 95.15%)	pp, us	P, S
*Penicillium pancosmium*	BEOFB0114000	PV871566 (100%, 0.0, 99.63%)	PV936452 (99%, 0.0, 100.00%)	us	S
*Penicillium solitum*	BEOFB0110903	PV871533 (100%, 0.0, 99.80%)	PV936447 (99%, 0.0, 100.00%)	en	Sy
*Penicillium vulpinum*	BEOFB0113900	PV871561 (100%, 0.0, 100.00%)	PX099059 (100%, 0.0, 100.00%)	us	S
*Prillingera fragicola* *	BEOFB1060000	PV871548 (100%, 0.0, 99.79%)	-	ep, us	S, Sy
*Schizophyllum commune*	BEOFB0660005	PV871530 (100%, 0.0, 99.83%)	-	ap, ws, en	P, S
	BEOFB0660006	PV871531 (100%, 0.0, 99.30%)	-	ap, ws, en	P, S
Sporobolomyces roseus *	BEOFB0410001	PV871541 (100%, 0.0, 100.00%)	-	fp, ps	P, S
Thanatephorus cucumeris *	BEOFB0240003	PV871556 (100%, 0.0, 100.00%)	-	pp, en	P, Sy
*Trametes hirsuta*	BEOFB0840103	PV871565 (100%, 0.0, 99.66%)	-	pp, ws	P, S
*Trichotecium roseum*	BEOFB0150101	PV871555 (100%, 0.0, 99.82%)	-	pp	P

Legend: P—pathotroph; S—saprotroph; Sy—symbiotroph; ap—animal pathogen; en—endophyte; ep—epiphyte; fp—fungal parasite; pp—plant pathogen; ps—plant saprotroph; ss—soil saprotroph; us—undefined saprotroph; ws—wood saprotroph; na—not applicable, no overlap for the target region; * first record for cave environment according to Savković et al. [4].

## Data Availability

The datasets generated and analyzed during this study are included in the article. Further inquiries can be directed to the corresponding authors.
